# Investigating the Interplay: Periodontal Disease and Type 1 Diabetes Mellitus—A Comprehensive Review of Clinical Studies

**DOI:** 10.3390/ijms25137299

**Published:** 2024-07-02

**Authors:** Stefania Vlachou, Alexandre Loumé, Catherine Giannopoulou, Evangelos Papathanasiou, Alkisti Zekeridou

**Affiliations:** 1 Division of Regenerative Dental Medicine and Periodontology, University Clinics of Dental Medicine, University of Geneva, Rue Michel-Servet 1, 1211 Geneva, Switzerland; stefania.vlachou@unige.ch (S.V.); alexandre.loume@unige.ch (A.L.); ekaterini.giannopoulou@unige.ch (C.G.); 2 Department of Periodontology, Tufts University School of Dental Medicine, One Kneeland Street, Boston, MA 02111, USA; evangelos.papathanasiou@tufts.edu

**Keywords:** diabetes mellitus, periodontitis, type 1 diabetes mellitus, molecular mechanisms, cellular mechanisms, oral microbiota, host immune response, oxidative stress, periodontal disease

## Abstract

Diabetes mellitus (DM) poses a significant challenge to global health, with its prevalence projected to rise dramatically by 2045. This narrative review explores the bidirectional relationship between periodontitis (PD) and type 1 diabetes mellitus (T1DM), focusing on cellular and molecular mechanisms derived from the interplay between oral microbiota and the host immune response. A comprehensive search of studies published between 2008 and 2023 was conducted to elucidate the association between these two diseases. Preclinical and clinical evidence suggests a bidirectional relationship, with individuals with T1DM exhibiting heightened susceptibility to periodontitis, and vice versa. The review includes recent findings from human clinical studies, revealing variations in oral microbiota composition in T1DM patients, including increases in certain pathogenic species such as *Porphyromonas gingivalis*, *Prevotella intermedia*, and *Aggregatibacter actinomycetemcomitans*, along with shifts in microbial diversity and abundance. Molecular mechanisms underlying this association involve oxidative stress and dysregulated host immune responses, mediated by inflammatory cytokines such as IL-6, IL-8, and MMPs. Furthermore, disruptions in bone turnover markers, such as RANKL and OPG, contribute to periodontal complications in T1DM patients. While preventive measures to manage periodontal complications in T1DM patients may improve overall health outcomes, further research is needed to understand the intricate interactions between oral microbiota, host response, periodontal disease, and systemic health in this population.

## 1. Introduction

Diabetes mellitus (DM) is identified as a challenge to global health, impacting a substantial segment of the global population. According to projections by the International Diabetes Federation (IDF), the prevalence of diabetes is expected to rise significantly by 2045; it is estimated that 1 in 8 adults, approximately 783 million people, will have DM. The two primary types of diabetes mellitus are type 1 (T1DM) and type 2 (T2DM). T1DM, also called juvenile-onset or insulin-dependent diabetes, is characterized by the autoimmune destruction of pancreatic beta-cells resulting in insulin deficiency, which predominantly affects young individuals [1]. T2DM, also called adult-onset or non-insulin-dependent diabetes, is characterized by insulin resistance.

There is compelling evidence suggesting a bidirectional relationship between periodontitis (PD) and DM, both being chronic inflammatory conditions. Individuals with diabetes demonstrate a higher prevalence of periodontitis, and conversely, those with periodontitis are more prone to diabetes-related complications [2,3,4]. While the relationship between periodontitis and type 2 diabetes mellitus is more extensively studied in the literature, there is a growing interest in understanding its connection with type 1 diabetes mellitus as well [5].

This narrative review aims to elucidate the association between periodontitis and T1DM, focusing on cellular and molecular mechanisms, with particular emphasis on the role of oral microbiota and immune responses. Amidst the increasing focus on T1DM, this review provides a pioneering investigation, greatly enriching our comprehension of the intricate interplay between periodontitis and T1DM. We conducted a comprehensive review of articles pertaining to these two diseases, published in English between 2008 and 2023. Our search utilized the National Library of Medicine, PubMed search engine, employing specific search pathways such as: ((hyperglycemia) AND (diabetes mellitus type I)) AND (modifications in microbiota), type 1 diabetes and periodont* and inflammation, and type 1 diabetes and periodont* and immune response. Few older reference articles frequently cited in the literature were included. The review included all clinical studies published within this timeframe, focusing on the correlation and mechanisms linking periodontal disease and T1DM. All authors screened the articles and categorized them into two groups: studies on microbiological findings (22 studies, 6 of which were published before 2008 but frequently cited in recent articles) and studies on the host immune response (9 studies). 

## 2. Type 1 Diabetes Mellitus

T1DM is the most common autoimmune disease in young patients. This pathology is characterized by the dysregulation of glucose metabolism, attributed to the gradual autoimmune destruction of pancreatic beta cells. Consequently, individuals with T1DM exhibit insulin deficiency coupled with hyperglycemia [1]. According to estimates provided by the American Diabetes Association, T1DM accounts for approximately 5–10% of all diabetes cases. Clinical manifestations commonly associated with T1DM include polydipsia, polyphagia, and polyuria [1].

The global incidence of T1DM has displayed a consistent upward trend, with rates escalating by 2–5% across various regions worldwide. Significantly considerable heterogeneity in diagnosis exists between different geographic locales or continents [6,7,8]. While the epidemiology of childhood-onset T1DM is extensively documented and routinely updated in the IDF Diabetes Atlas, the landscape of adult-onset T1DM remains less elucidated. This knowledge gap can be attributed to historical biases favoring childhood-onset cases, challenges associated with distinguishing adult-onset T1DM from T2DM, and the absence of comprehensive national diabetes registries encompassing T1DM incidence across all age groups [6,9]. Nonetheless, it is acknowledged that T1DM predominantly manifests in childhood but can occur at any age.

A recent systematic review conducted by Harding et al. (2022) [10] aimed to evaluate the incidence of adult-onset T1DM (occurring in individuals over 20 years of age) across 32 countries and regions. The findings of this review underscored a notable burden of adult-onset T1DM incidence, thereby emphasizing the urgent need to enhance both the quality and quantity of information pertaining to adult-onset T1DM, particularly in low- and middle-income countries.

## 3. Clinical Phenotype of T1DM

T1DM presents a multifaceted clinical phenotype predicated on two key assumptions. Firstly, the onset of T1DM is characterized by evidence of islet-directed autoimmunity preceding the manifestation of dysglycemia or hyperglycemia. Secondly, the pathogenesis of T1DM encompasses diverse pathways leading to beta cell destruction, influenced by variables such as age of onset, genetic predisposition, pancreatic pathology, metabolic dysregulation, insulin secretion dynamics, diabetic complications, and therapeutic responses [11,12].

The clinical diagnosis of T1DM relies on two primary features: insulin deficiency necessitating exogenous insulin therapy and the presence of islet-directed autoantibodies. These criteria serve as pivotal markers guiding the clinical assessment of the disease [13].

T1DM progresses through three discernible stages. The initial stage is characterized by the detection of autoantibodies in the absence of hyperglycemia, defining the prediabetic phase. Subsequently, the second stage ensues with the development of hyperglycemia. Finally, overt diabetes manifests in the third stage, marked by the emergence of clinical symptoms revealing the diabetic process [14].

## 4. Pathogenesis of Type 1 Diabetes Mellitus

Autoantibodies serve as vital biological markers for autoimmune diabetes, albeit their direct involvement in beta-cell destruction is limited. In children under 5 years old who develop diabetes, these autoantibodies are detected in nearly 100% of cases. They often serve as predictive indicators for diabetes occurrence in first-degree relatives of T1DM patients or in newborns from T1DM parents. Their detection typically precedes disease onset by months or even years, varying with the age of onset. Notably, the presence of two or more antibodies before the age of 3 correlates with a 75% risk of developing T1DM within 10 years, while the presence of all four antibodies indicates a 100% risk over a 20-year follow-up period [15].

Contemporary understanding characterizes T1DM as a multifactorial and heterogeneous disease, with diverse trajectories among patients. While the precise mechanisms remain elusive, genetic, and environmental factors are implicated, suggesting an intricate interplay [16]. Among environmental influences, infections, probiotics, micronutrients, and microbiota have emerged as significant factors in either triggering or exacerbating the disease process [17,18].

Current consensus within the scientific community underscores the autoimmune response to beta cells as precipitated by a myriad of environmental triggers in genetically predisposed individuals. Notably, variations in T1DM incidence among countries likely reflect disparities in susceptibility genetic loci rather than environmental exposures. However, the declining occurrence of high-risk Human leukocyte antigen (HLA) alleles in T1DM cases suggests a pivotal role for gene-environment interactions [19]. The initiation of beta-cell autoimmunity involves diverse environmental factors and gene-environment interactions, mediated by the activation of autoreactive CD4+ helper T cells and CD8+ cytotoxic T cells, ultimately leading to beta-cell apoptosis [13,20].

**Figure 1:**
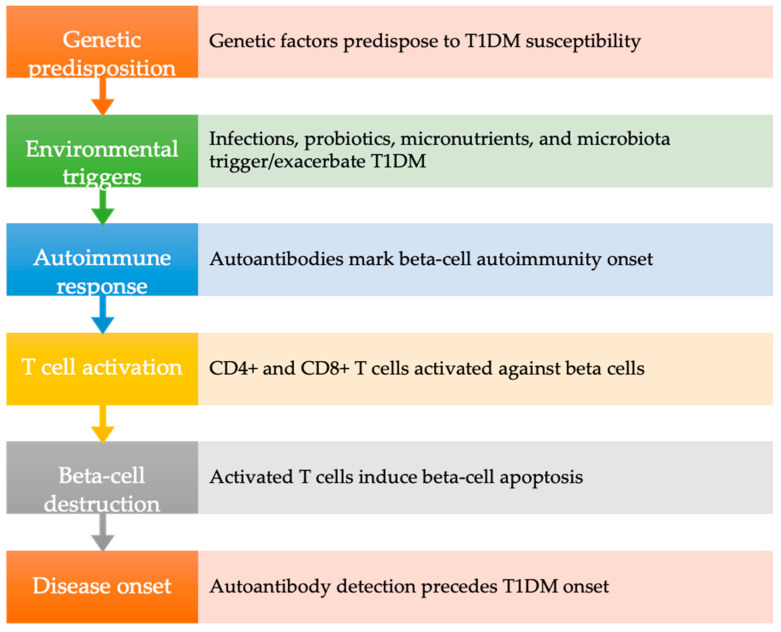
Pathogenesis of type 1 diabetes mellitus. T1DM: type 1 diabetes mellitus.

The subsequent chapter delves into evidence discussing the association between T1DM and periodontitis, incorporating findings from both animal and human studies (Figure 1).

## 5. Preclinical and Clinical Evidence

Animal studies have utilized rodent models, including chemically induced and genetically caused T1DM, to investigate the bidirectional relationship between periodontitis and T1DM. Streptozotocin and alloxan can chemically induce an immune-mediated form of T1DM in rodents, while the non-obese diabetic mouse develops an autoimmune affection in the pancreas resembling human T1DM development [21]. Studies have revealed that periodontal bone loss in T1DM rats is three times higher than in normal rats [22,23] corroborating findings from human studies on the impact of diabetes on the periodontium. However, few rodent studies have explored the consequences of periodontitis on diabetes.

In human studies the association between the two conditions was explored at the level of prevalence, clinical periodontal parameters, and metabolic control. For instance, a cross-sectional study found that periodontitis affected 15.0% of controls and 57.9% of diabetic patients, with severe periodontitis more prevalent in poorly controlled diabetic patients [24]. Another study reported that almost one in five T1DM patients also suffer from periodontal disease [25]. Clinically, diabetic subjects exhibit more plaque, gingival inflammation, and attachment loss compared to controls [26,27]. Additionally, poor glycemic control is associated with worse periodontal clinical parameters [28]. These parameters are strongly correlated with diabetes duration and HbA1c levels [29]. Moreover, T1DM patients exhibit not only increased susceptibility to periodontal diseases, but also to dental caries particularly when metabolic control is poor [26,30]. Meta-analyses have consistently shown higher prevalence and severity of periodontal disease in diabetic patients, with elevated HbA1c levels linked to worse clinical parameters [2,31,32,33]. Interestingly, even patients with relatively good metabolic control, such as those treated with continuous subcutaneous insulin infusion, show a higher prevalence of mild gingivitis compared to non-diabetic individuals [34].

The effect of periodontal treatment on glycemic control in T1DM patients with periodontitis has equally been studied. While periodontal health improved after treatment, no significant effect on glycemic control was observed [35,36]. Nevertheless, providing periodontal care to diabetic patients remains important.

It is evident that the clinical periodontal status of patients with type 1 diabetes is characterized by a heightened susceptibility to periodontal disease, manifesting as gingival inflammation, periodontal pocket formation, and alveolar bone resorption [28]. As mentioned before, longitudinal studies indicate a progression of periodontal disease in diabetic individuals, with a significant correlation between glycemic control and periodontal health [37]. Therapeutically, managing periodontal disease in patients with type 1 diabetes requires a comprehensive approach that integrates periodontal treatment modalities with meticulous glycemic control strategies. Effective management not only aims to arrest periodontal disease progression but also potentially mitigates the systemic complications associated with both diabetes and periodontitis. Effective management of periodontal health in T1DM patients necessitates regular monitoring, stringent glycemic control, interdisciplinary collaboration between healthcare providers, and personalized treatment plans. Emphasizing patient education on the critical connection between diabetes and periodontal disease can also play a pivotal role in mitigating these adverse outcomes. Preventive measures tailored to managing periodontal complications and the use of host modulatory agents in individuals with T1DM can help to improve their overall oral and systemic health outcomes [38].

## 6. Type 1 Diabetes Mellitus and Oral Microbiota

Environmental factors have been recognized as significant contributors to the onset and progression of T1DM, with emerging attention on the role of the gut microbiota as evidenced by “The Environmental Determinants of Diabetes in the Young Study” [39]. It is hypothesized that the gut microbiome influences T1DM risk through mechanisms such as altered intestinal permeability, modulation of the gut immune system, and molecular mimicry. Animal studies have demonstrated associations between gut flora composition and autoimmune diabetes risk, with *lactobacillus* and *bifidobacterium* linked to diabetes resistance, and *bacteroides* associated with susceptibility [40].

Similarly, human studies reveal disparities in gut microbiota between diabetic and healthy individuals, with T1DM patients exhibiting less diverse and potentially harmful organisms [41]. These variations are influenced by factors such as antibiotic use, dietary components, hygiene practices, and geographical location, all which impact microbiome composition.

Clinical investigations on the gut microbiome consistently report increased *Bacteroides species* in T1DM subjects compared to controls, alongside increases in *Bacilli*, *Enterobacteriaceae*, *Streptococcus*, *Ruminococcus*, and *Prevotella*, with decreases noted for *Bifidobacteria*, *Butyrate-producing bacteria*, *Haemophilus*, *Veillonella*, and occasionally *Prevotella*. Recently, Abudqwider et al. (2023) conducted a systematic review exploring the interplay between the gut microbiome, inflammation, and blood glucose parameters in T1DM patients, finding correlations between HbA1c levels and specific microbial abundances, with *Prevotella*, *Faecalibacterium*, and *Ruminococcaceae* negatively correlated, and *Dorea formicigenerans*, *Bacteroidetes*, *Lacrobacillales*, and *Bacteroides* positively correlated. *Bifidobacteria* showed a negative correlation with fasting blood glucose levels [42].

These studies collectively indicate that T1DM is associated with gut dysbiosis and increased intestinal permeability, suggesting modulation of the gut microbiome as a novel therapeutic avenue.

Several efforts have been made to identify if similar modifications appear in the oral microbiome based on the hypothesis that various factors, including oral and systemic diseases like diabetes, may contribute to changes in the composition of the oral microbiome [43,44]. Understanding the microbial variations is particularly complex in T1DM due to the prolonged period between initial β-pancreatic cell damage and clinical disease manifestation [45].

Studies on the oral microbial composition in individuals with T1DM (insulin-dependent diabetes mellitus), have revealed variations in the quantity and quality of bacterial species compared to healthy controls. For instance, Mashimo et al. (1983) observed a microflora predominantly composed of *Capnocytophaga species* and *anaerobic vibrios* in individuals with insulin-dependent diabetes mellitus (IDDM) [46]. In contrast, Sastrowijoto et al. (1989) reported low levels of *Capnocytophaga species* and elevated levels of *Porphyromonas gingivalis*, *Prevotella intermedia*, and *Aggregatibacter actinomycetemcomitans* in IDDM patients [47].

Some studies demonstrated a significant increase in *Gram-negative rods* and *fusiforms* in the subgingival microbial composition of T1DM patients, influenced by age, duration of diabetes, or metabolic control, as indicated by HbA1c scores [48], while others found a significantly higher percentage of *Prevotella intermedia* in periodontal sites with deep pockets among poorly controlled T1DM patients [49]. Examining poorly controlled T1DM patients, they noted elevated levels of pathogens at diseased sites, including *Prevotella intermedia*, *Prevotella melaninogenica*, *Campylobacter gracilis*, *Eikenella corrodens*, *Fusobacterium nucleatum*, and *Campylobacter rectus*, with a significantly higher percentage of *Prevotella intermedia* noted at sites with deep pockets and attachment loss. Contrary, another study also compared the periodontal condition and subgingival microbial composition of insulin-dependent juvenile diabetic patients with their non-diabetic siblings, finding no statistically significant differences for the tested microorganisms [50].

Recent research has further delved into the relationship between glycemic control, oral microbiota composition, and periodontal health in children with T1DM. Sjodin et al. (2012) compared the microbiota of young adults with T1DM since childhood to an age- and sex-matched non-diabetic control group, observing differences in the prevalence of specific bacteria, though not significant [51]. Diabetics with poor metabolic control exhibited a lower frequency of certain bacteria, emphasizing potential distinctions in microbial composition [51].

Others expanded this investigation by evaluating the subgingival microflora in both insulin-dependent and non-insulin-dependent diabetic patients with periodontitis. Although predominant organisms were identified in the insulin-dependent diabetic group, the study failed to establish statistically significant differences in subgingival microflora between diabetic and healthy individuals [52].

Other studies found no significant difference in the prevalence of putative periodontopathic bacteria between T1DM and healthy children, challenging the notion of an increased risk of periodontitis associated with specific bacterial species in T1DM subjects [53]. Similarly, Lalla et al. (2006), suggested similar subgingival infection patterns between individuals with T1DM and healthy controls, particularly under controlled periodontal disease severity conditions [54]. Accordingly, Singh-Hüsgen et al. (2016) demonstrated that the oral microflora of diabetic children did not differ significantly from that of healthy subjects, challenging previous notions of altered host responses in diabetics leading to increased tissue destruction [55].

On the contrary, some investigations on the interplay between diabetes, periodontal parameters, and microbiota, revealed variations in certain microorganisms between periodontitis patients with and without diabetes [56,57,58] with specific bacteria, such as the *F. nucleatum* and the *Capnocytophaga* spp. showing strong associations with diabetes [59].

Recently Mahalakshimi et al. (2019) evaluated the risk of periodontitis associated with specific bacteria in T1DM children, revealing differences in gingival health but no statistically significant association with bacterial prevalence [60]. Other studies continued to explore the correlation between HbA1c levels, oral microbiota, and specific bacterial species, uncovering associations with microbial diversity and periodontal disease in T1DM subjects [61,62].

Many characterized the oral microbiota in children and adults with T1DM, identifying distinctions in bacterial abundance, microbial diversity, and specific genera, providing a more comprehensive understanding of the oral microbiome in the context of T1DM [34,43,63].

In a recent clinical trial, Carelli et al. (2023) explored the association between oral microbiota, dental and periodontal diseases, and glycemic control in T1DM children and adolescents, observing consistent presence of specific bacterial species and associations with poor glycemic control, adverse metabolic outcomes, and oral hygiene practices [64].

The cross-sectional study by Selway et al. (2023) further underlined the complexity of the relationship between oral microbiota, periodontal health, and systemic factors in children with T1D, with microbial diversity influenced by periodontal risk markers and familial history of hyperlipidemia. This emphasizes the multifaceted nature of the oral microbiota in this population, suggesting a potential role of *non-Porphyromonas species*, such as *Prevotella*, in contributing to periodontal disease in children with a family history of hyperlipidemia [65]. This emphasizes the multifaceted nature of the oral microbiota in this population, suggesting a potential role of *non-Porphyromonas species*, such as *Prevotella*, in contributing to periodontal disease in children with a family history of hyperlipidemia. The disruption of microbial ecosystems in these children may involve putative pathogens that contribute to periodontitis and cardiovascular risk factors in subjects.

The current understanding of bacterial-host interactions in periodontal disease related to T1DM is still limited, emphasizing the necessity for additional research to comprehend the intricate connections between systemic health and periodontitis (Table 1). Furthermore, exploring potential connections between oral microbiota and diabetes during the latent phase offers opportunities for early intervention and potentially delaying disease onset. The main microbiological findings are summarized in Table 2.

## 7. Type I Diabetes Mellitus and Host Immune Response

The contribution of a dysregulated exaggerated host immune/inflammatory response in periodontitis is clear. Although a large number of studies describing the bi-directional relationship between periodontitis and T2DM is available in the literature [66] the potential mechanisms underlying the possible association between T1DM and periodontal diseases remain unclear. While exploring the intricate relationship between T1DM and PD, it becomes evident that the host inflammatory and immune response serve as a key player driving the progression of both diseases. However, it is increasingly recognized that periodontal pathologies may represent complications of T1DM, sharing common pathogenic mechanisms with other macro- and micro-vascular complications of diabetes, such as retinopathy and nephropathy [67].

The host immune response involves inflammation of gingiva and can be modulated by several host-related factors including diabetes mellitus, smoking, genetics and stress [68]. The immune response and inflammation emerge as central links between autoimmune disorders like T1DM and PD. Notably, hyperglycemia stands out as a crucial risk factor, triggering oxidative stress and inflammation that accelerate tissue dysfunction in the periodontium. Several human clinical studies have been undertaken to explore potential immunological pathways connecting these two diseases (Table 3).

## 8. Oxidative Stress

Oxidative stress stands as a pivotal factor in the pathogenesis of diabetes mellitus and periodontal diseases, often serving as a reliable marker for screening diabetes-related periodontal dysregulation [69]. The cascade of hyperglycemia prompts an upsurge in Reactive Oxygen Species (ROS), culminating in structural alterations within proteins, nucleic acids, and lipids, thereby disrupting cellular functionality [78]. Notably, heightened levels of oxidative stress markers have been detected in the Gingival Crevicular Fluid (GCF), saliva, and serum of T1DM patients, showing positive correlations with glycated hemoglobin [69]. Furthermore, oral hygiene education coupled with professional scaling has demonstrated a notable reduction in oxidative stress markers among T1DM patients three months post-periodontal treatment [69]. However, a recent study among Thai adolescents and young adults with T1DM showed no disparity in salivary oxidative stress biomarkers when compared to their healthy counterparts [70]. Nevertheless, salivary total oxidative status levels were linked to both diabetes status and the extent of gingival inflammation, warranting further exploration through clinical studies encompassing varying degrees of periodontal disease [70]. Additionally, Lipski et al. (2021) revealed that reinforcing proper oral hygiene with antibacterial dentifrices notably reduced specific salivary oxidative stress biomarkers in young T1DM patients with gingivitis [79]. Moreover, analyses of GCF microbiology and metabolomics in adults with T1DM undergoing continuous subcutaneous insulin infusion suggested early alterations in the GCF microbiome and metabolite concentrations, potentially attributed to increased oxidative stress markers, thus urging further investigation [34].

## 9. Host Immune Markers

The interplay between high blood glucose levels and sustained chronic inflammatory mediator secretion significantly contributes to an exaggerated periodontal response in individuals with T1DM. Studies investigating the increase in host immune markers in patients with T1DM and periodontal diseases are expected to shed further light into the processes linking T1DM and periodontitis and provide significant value in understanding better the mechanisms behind diabetic complications and introducing novel therapeutic targets. Elevated plasma levels of IL-8 were found in patients with T1DM and periodontitis [71,80] but also in patients with T1DM independently on their periodontal status possibly associated with high glucose-induced oxidative stress. Furthermore, serum IL-6 levels have shown positive correlations with the extent of periodontal inflammation in T1DM patients, while serum high-density lipoprotein (HDL) cholesterol levels exhibited negative correlations [72,81]. Notably, heightened serum IL-6 levels post-periodontal therapy in T1DM patients were linked to poorer periodontal healing responses, suggesting a potential modulation of the host immune response by IL-6 in T1DM patients [81]. Additionally, increased GCF levels of IL-1β and MMP-9 have been reported in T1DM patients during experimental gingivitis, unrelated to microbial differences [73]. Conversely, Sereti et al. (2021) found no differences in GCF levels of IL-8, MMP-8, and advanced glycation end products (AGEs) between T1DM and non-diabetic individuals [82]. Despite the small sample size, gingival biopsies from adult T1DM patients with aggressive periodontitis exhibited increased expression of MMP-7, -8, -9, and -13 compared to patients without diabetes, underscoring the importance of early periodontal therapy in T1DM [83].

Controversial results have been reported regarding IL-18 levels in children with T1DM due to limitations in collecting samples from an adequate number of patients in order to draw safe conclusions. GCF IL-18 levels were found higher in children with T1DM (n = 30) and gingivitis compared to healthy children with gingivitis (n = 13) [84], while in a larger study GCF IL-18 levels were similar between diabetic (n = 44) and healthy children (n = 44) with gingivitis [85]. While most of the studies compared levels of inflammatory cytokines between diabetic and systemically healthy patients, one very interesting study compared immune markers between T1DM and T2DM with periodontitis [74]. Unexpectedly, GCF IL-1β and TNF-α levels were higher in T1DM periodontitis patients compared to T2DM periodontitis ones. The authors of this study also showed that GCF IL-1β and TNF-α levels were negatively correlated with diabetes duration are higher in cases of recent onset of the disease highlighting the need for periodontal therapy at the early stages of T1DM development. Good metabolic control affects periodontal inflammation in diabetic patients. Indeed, T1DM patients post simultaneous pancreas and kidney transplantation displayed lower GCF levels of inflammatory markers and reduced intensity of periodontitis compared to insulin-treated kidney recipients [75].

Salivary IgA levels were lower in T1DM patients with diabetic neuropathy compared to healthy individuals, offering a non-invasive method for assessing the risk of developing diabetic neuropathy [86]. Furthermore, a case–control study revealed increased salivary IL-17 levels in diabetic children, highlighting potential immune dysregulation in T1DM [87]. Additionally, Yilmaz et al. (2023) demonstrated altered salivary concentrations of macrophage activation-related chemokines and MAPKK-degrading proteolytic activity in T1DM patients showing higher levels of monokine induced by interferon gamma (MIG) and macrophage inflammatory protein-1 alpha (MIP-1α) in saliva of T1DM patients, underscoring the impact of T1DM on the host immune response [88].

Studies exploring bone markers in T1DM patients with periodontitis have revealed intriguing findings. While T1DM patients with periodontitis exhibited lower plasma RANKL:OPG ratios compared to non-diabetic counterparts, they displayed higher serum OPG levels, suggesting impaired bone turnover in T1DM patients during periods of acute periodontitis [76]. Similarly, Antonoglou et al. (2013) reported increased serum OPG levels in T1DM patients, positively correlating with the severity of periodontitis, emphasizing the need for further investigation into the role of OPG in T1DM-associated periodontal diseases [89]. Furthermore, Chairatnathrongporn et al. (2022) observed increased RANKL and RANKL:OPG ratio alongside decreased OPG gene expression levels in saliva of T1DM patients compared to healthy individuals, advocating for more extensive analysis of bone markers in oral fluids to elucidate their role in T1DM and periodontal diseases [77].

While current evidence underscores the intricate interplay between oxidative stress, host immune markers, and periodontal diseases in T1DM patients, further large-scale clinical studies are warranted to validate differences in inflammatory mediators and bone markers between diabetic and non-diabetic individuals across various stages of periodontal diseases.

## 10. General Conclusions and Future Suggestions

The dynamic interplay between PD and T1DM highlights the significance of comprehensive dental care in managing diabetes mellitus. It is evident from current research that both diseases share common pathogenic mechanisms. A key feature and limitation of this review is its narrative approach, distinct from the systematic methodology commonly used in systematic reviews. Consequently, the narrative format employed here may introduce subjectivity into the selection of included studies and their interpretation. Unlike systematic reviews, our aim is to provide valuable insights into the interplay between the two diseases, rather than incorporate quantitative synthesis techniques like meta-analysis. Recognizing these differences in approach is crucial, as they can shape the interpretation and implications of the findings.

Conducting longitudinal assessments to delineate close interactions between oral microbiota, host response, periodontal disease, and systemic health in T1DM patients could help to develop targeted interventions aimed to mitigate the impact of periodontitis on glycemic control and overall health in individuals with T1DM.

## Figures and Tables

**Table 1:** Clinical studies on microbiological findings in diabetes mellitus type I and periodontal disease.

References	Type of Study	Methodology	Types of Samples & Microbiological Analysis	Results	Conclusions
Arangann et al., 2013 [53]	Comparative case–control clinical study	Dental and medical examination Subgingival plaque sampling from permanent first molars	Polymerase chain reaction assay for 6S rRNA gene detection	No significant statistical difference regarding the prevalence of *P. Gingivalis, T. Denticola*, and *A. actinomycetemcomitans* among T1DM and healthy children *T. Forsythia* was less prevalent in diabetic children compared to healthy children	Similar microbiologic findings in T1DM patients and healthy children
Carelli et al., 2023 [64]	Cross-sectional study	HbA1c Continuous glucose monitoring metrics (GCM) of glycemic control and glucose variability Saliva sampling	DNA extraction Bacterial culture-based analysis PCR	*Actinomyces* spp., *A. actinomycetemcomitans*, *P. intermedia*, and *Lactobacillus* spp. in all subjects *S. mutans* present in approximately 49.4% of samples Higher presence of *S. mutans* and *Veillonella* spp. in subjects with poorer glycemic control (HbA1c, %TIR, %TAR). Association observed even after adjusting for age, sex, and hygiene habits Virtuous habits (toothbrush changes, professional oral hygiene) negatively correlated with *T. forsythia*, *T. denticola*, and *P. gingivalis*	Importance of glycemic control and regular oral hygiene in preventing oral microbiota linked to dental and periodontal issues in individuals with T1DM since childhood
Castrillon CA et al., 2015 [58]	Comparative case–control clinical study	Periodontal examination Subgingival plaque samples from the three deepest sites	DNA extraction by silica	Diabetic patients showed significantly higher levels of periodontal attachment loss than non-diabetic patients *A. actinomycetemcomitans* was more prevalent in diabetic patients with periodontitis compared to systemically healthy patients without periodontitis *P. gingivalis* was associated with periodontitis in non-*diabetic patients*, while *A. actinomycetemcomitans* was linked to periodontitis in diabetic patients	Subgingival microbiota differences exist between diabetic and non-diabetic patients *P. gingivalis* and *A. actinomycetemcomitans* are associated with periodontitis in non-diabetic and diabetic patients, respectively
Chakraborty et al., 2021 [62]	Cross-sectional study	Gingival plaque sampling	Culture based microbial identification and biofilm assay	Higher microbial diversity in diabetes with periodontal disease group *S. warneri* found only in diabetes with periodontal disease group Higher incidence of *S. vitulinus, S. sanguinis*, and *P. aeruginosa* in diabetes with periodontal disease group Strong positive correlation between poor glycemic control and biofilm formation in both diabetes with and without periodontal disease	T1DM children with worse glycemic control, especially with periodontitis, showed increased biofilm formation and microbial diversity
Duque et al., 2017 [57]	Comparative case–control clinical study	Periodontal status Glycemic and lipid profiles Subgingival plaque samples from periodontal sites Blood samples for IL-1b, TNF- and IL-6	PCR ELISA	Similar periodontal conditions in T1DM and non-diabetic patients Higher lipid parameters in DM group among patients with gingivitis Increased prevalence of *C. sputigena* and *C. ochracea* in periodontal sites of DM children Limited detection of “Red complex” bacteria in both groups Frequent occurrence of *F. nucleatum* and *C. rectus* Comparable levels of TNF-α and IL-6 in both groups	Gingivitis in type 1 diabetes children associated with *C. sputigena* and *C. ochracea* Overall periodontal health and inflammatory markers similar between the two groups
Gregorczyk-Maga et al., 2023 [34]	Pilot study	Samples from the mucosa of the buccal and soft palate, tongue, palatal and buccal dental surfaces, and gingival pockets in adult patients with type 1 diabetes mellitus undergoing treatment with insulin pump therapy	Microbiologival cultures with dilution method or qualitative culture method	Dominant oral microbiota included *Streptococcus* and *Neisseria* Low incidence of cariogenic *S. mutans*, *Lactobacillus*, and periodontal pathogens like *Prevotella* Significant differences in CFU counts observed between mucosal and dental surface sites for all strains, *Gram-positive*, *Staphylococci*, *Streptococci*, and *Streptococcus oralis* strain *Candida species* were rare Mucosal sites exhibited lower diversity and bacterial counts	Adult T1DM patients treated with insulin pomp, distinct differences in oral microbiota were observed in specific niches Identified optimal sampling sites for oral microflora: buccal and palatal mucosa, dental surface, and gingival pockets
Jensen et al., 2021 [61]	Cross-sectional study	Dental and periodontal examination Buccal and gingival bacterial sampling	16S rRNA sequencing	49% had early markers of periodontal disease Positive correlations between HbA1c and plaque index, gingival index, bleeding on probing, and periodontal pocket depth > 3 mm A 1% increase in HbA1c associated with a 25% increase in bleeding on probing and a 54% increase in sites with pocket depth > 3 mm Higher HbA1c independently related to increased phylogenetic alpha diversity and compositional variation in gingival microbiota Brushing frequency, plaque index, and gingival index affected microbiota composition, independent of HbA1c	Relationship between less favorable glycemic control and increased early markers of periodontal disease Glycemic control is linked to the complexity and richness of plaque microbiota, with diversity increasing as HbA1c levels rise
Kumar et al., 2012 [52]	Comparative parallel group clinical study	Dental and periodontal examination Sub gingival plaque sampling	Bacterial cultures with ‘standard loop semiquantitative method’.	No statistical difference between the three groups (insulin-dependent, non-insulin dependent diabetics and non-diabetic periodontitis patients)	The microbial flora of the periodontitis patients is not influenced by their diabetic status
Lalla et al., 2006 [54]	Comparative case–control clinical study	Periodontal examination Blood samples Subgingival plaque sampling	DNA–DNA hybridization Cytokine multiplex analyses Checkboard immunoblotting	Elevated levels of *E. nodatum* in diabetic patients among the i IgG titres showed no significant differences between diabetic and non-diabetic groups, both before and after adjustments for microbial load Diabetic patients exhibited higher serum levels of soluble E-selectin, vascular cell adhesion molecule-1, and adiponectin Diabetic patients had lower levels of plasminogen activator inhibitor-1	Controlling for the severity of periodontal disease, both T1DM patients and non-diabetic controls displayed comparable subgingival infection patterns Serum antibody responses were similar between diabetic and non-diabetic groups after adjusting for periodontal disease severity
Mahalakshmi et al., 2019 [60]	Comparative case–control clinical study	Periodontal examination Subgingival plaque sampling	PCR	No statistically significant difference in the prevalence of *C. rectus*, *E. corrodens*, *P. intermedia*, *P. nigrescens* between type 1 diabetic and healthy children	Negative correlation of T1DM with periodontitis in association to 4 periopathogenic bacteria
Mandell et al., 1992 [49]	Cross-sectional study	Periodontal examination Subgingival plaque sampling	Bacterial cultures	Elevated levels of periodontal pathogens at diseased sites, including *P. intermedia*, *P. melaninogenica* spp., and others Higher prevalence of *P. intermedia* and *melaninogenica* spp., and *Campylobacter rectus* at diseased sites Significantly higher percentage of *P. intermedia* at sites with deep pockets and attachment loss	Correlation observed between periodontal disease in insulin dependent diabetes patients (T1DM) and increased levels of specific pathogens
Mashimo et al., 1983 [46]	Cross-sectional study	Periodontal examination Subgingival plaque sampling	Bacterial cultures Immunofluorescence microscopy ELISA	Predominance of *Capnocytophaga* and *anaerobic vibrios* in cultivable microflora of individuals with insulin-dependent diabetes and periodontitis Presence of *A. actinomycetemcomitans* in some individuals with insulin-dependent diabetes (T1DM) Distinct subgingival flora in individuals with insulin-dependent diabetes and periodontitis compared to patients periodontitis Periodontitis lesions in nondiabetic adults often contain black-pigmented *Bacteroides* such as *gingivalis* or *melaninogenicus subspecies intermedius* Antibiotic susceptibility patterns suggest the potential effectiveness of penicillin, tetracycline, or its analogs like minocycline against the predominant cultivable microflora in periodontal lesions of individuals with with insulin-dependent diabetes (T1DM)	The subgingival organisms identified in periodontal lesions of individuals with insulin-dependent diabetes (T1DM) exhibit quantitative distinctions from those observed in cases of periodontitis
Moskovitz et al., 2021 [43]	Comparative case–control clinical study	Periodontal examination Unstimulated saliva sampling	DNA extraction qPCR 16S rRNA library preparation Sequencing	Identified 105 genera and 211 species in salivary microbiome Abondant genera: *Streptococcus, Prevotella*, *Veillonella, Haemophilus*, *Neisseria* *Streptococcus* more abundant in type 1 diabetes children *Catonella*, *Fusobacterium*, *Mogibacterium* differed between healthy and T1DM subjects *Porphyromonas* and *Mogibacterium* correlated with salivary parameters	Salivary microbiome analysis revealed unique taxa in T1DM children Association between certain salivary genera and gut microbiome in T1DM patients
Olczak-Kowalczyk et al., 2015 [56]	Comparative parallel group clinical study	General medical examination Periodontal examination Oral bacterial swabs	Bacterial cultures Tests for enzymic profiles	*Candida* spp. detected in healthy and nephrotic syndrome/T1DM patients Oral candidiasis found in nephrotic syndrome and T1DM patients Gingivitis is more common in nephrotic syndrome/T1DM patients In diabetes, severity linked to blood glucose and glycated haemoglobin >8%	Gingivitis mainly caused by poor hygiene Severity associated with age, lipid disorders, and increased body mass *Candida* spp. may worsen plaque-related gingivitis in diabetes and immunosuppressed patients
Pachoński et al., 2021 [63]	Comparative case–control clinical study	Oral bacterial swabs	Bacterial cultures Sets of reagents for species identification	Statistically significant differences in the total number of isolated microorganisms were found between poorly controlled T1DM patients and healthy controls Statistically significant differences in the total number of isolated microorganisms were found between well controlled T1DM patients and healthy controls No statistically significant differences were observed in the number of isolated microorganisms between poorly and well controlled T1DM patients	Oral microbiome in T1DM children differs quantitatively and qualitatively from healthy children
Sakalauskiene et al., 2014 [59]	Comparative case–control clinical study	Periodontal and radiographic examination Blood samples Supra and subgingival plaque sampling	Bacterial cultures Molecular genetic assay	*F. nucleatum*, *Capnocytophaga* species, and *E. corrodens* were the most frequently identified bacteria in dental plaque samples *A. actinomycetemcomitans* was 40.7% less frequently identified in the diabetes type 1 group compared to the healthy group Periodontal disease was more pronounced IN T1DM patients The prevalence of periodontitis significantly increased in subjects with poorer metabolic control	The presence of two periodontal pathogens, *F. nucleatum* and *Capnocytophaga* spp., showed the strongest relationship with poorer metabolic control in T1DM patients and all clinical parameters of periodontal pathology
Sandholm et al., 1989 [48]	Comparative case–control clinical study	Subgingival plaque sampling	Bacterial cultures Microscopic observation	Significantly more *Gram-negative rods* and *fusiforms*, total *Gram-negative bacteria*, and *Rhodes-stained straight* and *curved rods* in patients compared to controls Controls have significantly more *Gram-positive* and *Gram-negative cocci*, *total Gram-positive bacteria*, and *Rhodes-stained fusiforms* than in insulin dependent diabetes patients Controls exhibit higher percentages of spirochetes and flagellated bacteria compared to in insulin dependent diabetes patients The distribution of morphotypes is not influenced by age, duration of diabetes, or metabolic control measured by HbA1c scores in patients	Diabetes patients exhibited lower proportions of cocci and total *Gram-positive bacteria* but higher proportions of periodontally more pathogenic forms, *Gram-negative rods*, and total *Gram-negative bacteria* compared to controls even if they had comparable hygiene
Sastrowijoto et al., 1989 [47]	Comparative case–control clinical study	General medical examination Periodontal examination Subgingival plaque sampling	Bacterial cultures and use of isolation and identification media	No significant difference in periodontal condition between patients in the poorly and well controlled T1DM patients Age of diabetic patients and duration of diabetes mellitus did not influence periodontal parameters Proportionally high percentages of cultivable bacteria were found in diseased periodontal pockets	Metabolic control had no direct effect on the periodontium The role of *Capnocytophaga species* in the pathogenesis of infectious periodontal disease in T1DM patients might be overestimated
Sbordone et al., 1995 [50]	Comparative case–control clinical study in siblings	General medical examination Periodontal examination Subgingival plaque sampling	Bacterial cultures and use of isolation and identification media Biochemical tests	No significant differences detected in any clinical and microbiological data	Limited distinctions were noted between individuals with T1DM and their healthy counterparts within this population
Selway et al., 2023 [65]	Post hoc cross-sectional study	Periodontal examination Medical and dental history Gingival swab samples	16S rRNA gene sequencing	Children with a family history of dyslipidemia exhibited distinct bacterial diversity and composition compared to those without a family history Differences were not observed in children with periodontal risk, irrespective of a family history of hyperlipidemia	In children diagnosed with T1DM, findings highlight an association between oral microbiota and two distinct exposure variables: familial history of hyperlipidemia and periodontal risk factors
Singh-Hüsgen et al., 2016 [42]	Cross-sectional study	Periodontal and dental examination Medical examination Supra and subgingival plaque sampling	PCR	A statistically significant difference in the decayed, missing, and filled surfaces (dmfs) index value was observed among the three groups (healthy, type 1 diabetes and phenylketonuria group) When comparing diabetics to healthy children, a small but statistically significant difference in the Periodontal Index (PBI) score was revealed Statistically significant differences were found in the counts of *Lactobacillus*, *Leptotrichia*, and *P. gingivalis* among the three groups	Diabetic children displayed a lower caries experience in their primary dentition but were found to have a slightly higher risk of developing periodontal disease
Sjodin et al., 2012 [51]	Comparative case–control clinical study	Periodontal, dental aand radiographic examination and history Medical examination and history Subgingival plaque sampling	Checkboard DNA-DNA hybridization	Periodontal health indicators, including probing pocket depths and marginal bone loss, were generally favorable in all patients The distribution of various microbiological species was similar between the study and control groups	Periodontal and microbiological status in young adults with insulin dependent diabetes (T1DM) does not differ significantly from that of healthy controls

T1DM: type 1 diabetes mellitus.

**Table 2:** Main microbiological findings in diabetes mellitus type I.

Main Microbiological Findings for T1DM
Gut microbiota influences T1DM risk
Animal studies link gut flora composition to T1DM risk
T1DM patients exhibit less diverse gut microbiota
Correlations found between HbA1c levels and specific microbial abundances in T1DM patients
Oral microbiome composition varies in individuals with T1DM
Variations in oral microbiota in T1DM patients observed compared to healthy controls
Increased Gram-negative rods and fusiforms found in subgingival microbial composition of T1DM patients

T1DM: type 1 diabetes mellitus.

**Table 3:** Clinical studies on host immune response in diabetes mellitus type I and periodontal disease.

Author	Type of Study	Methodology	Types of Samples & Immune Analysis	Results	Conclusions
Aral et al., 2017 [69]	Prospective case–control study	32 T1DM patients at diagnosis, and age- and gender-matched 36 systemically healthy children with (G) and without (H) gingivitis were enrolled for periodontal exam and oxidative stress markers. The diabetic patients who took insulin therapy (1.5 units/kg/day totally) and periodontal treatment (oral hygiene education with professional scaling) were re-evaluated after 3 months.	Total antioxidant status [64], total oxidant status (TOS), and oxidative stress index were measured in saliva, GCF and serum.	GCF, salivary, and serum oxidative stress index were higher in group T1DM compared to the other groups at baseline (*p* < 0.05). GCF, salivary, and serum oxidative stress index decreased 3 months after periodontal treatment in T1DM patients.	A substantial level of oxidative stress may occur in children with T1DM, with increased oxidative stress index in GCF, salivary, and serum samples.
Aroonrangsee et al., 2023 [70]	Cross-sectional case–control study	40 participants from 15–23 years old. 20 T1DM patients and 20 age- matched non-T1DM subjects were enrolled. An average HbA1c level of less than 8% was considered the cut-off between well-controlled and poorly-controlled T1DM. Unstimulated whole saliva was collected before clinical periodontal exam.	Salivary levels of OS biomarkers including malondialdehyde, protein carbonyl, total oxidant status (TOS), and total antioxidant capacity were determined using oxidative and antioxidative assays followed by spectrophotometric measurement at 375–532 nm.	T1DM was significantly associated with lower total oxidant status (TOS) in saliva (*p* < 0.05). Increased TOS levels were significantly correlated with BOP. No relationship was found between OS biomarkers and HbA1c levels.	Salivary total oxidant status (TOS) levels were related to both diabetes status and the extent of gingival inflammation.
Lappin et al., 2015 [71]	Cross-sectional study	104 participants in the study: 19 healthy volunteers, 23 patients with periodontitis, 28 patients with T1DM, and 34 patients with T1DM and periodontitis.	Levels of blood HbA1C were determined by high-performance liquid chromatography. Levels of IL-6, IL-8, and CXCL5 in plasma were determined by ELISA.	Higher levels of serum IL-8 in patients with T1DM and periodontitis compared to periodontitis alone. Higher serum levels of CXCL5 in T1DM patients compared to non-T1DM. No difference in plasma levels of IL-6 among the four groups.	Elevated plasma levels of IL-8 potentially contribute to the cross-susceptibility between periodontitis and T1DM.
Passoja et al., 2011 [72]	Prospective clinical study	80 subjects with T1DM (age 38.6 ± 12.3 y.o.) participated in the baseline study visit, while 58 subjects (age 39.5 ± 12.6 y.o.) completed the visit after periodontal therapy. Periodontal therapy included oral hygiene education, scaling and root planning and patients were re-evaluated 8 weeks after periodontal therapy. Periodontal exam and blood samples were drawn at the baseline and in the follow-up visits.	Serum IL-6 levels were measured using ELISA.	Significant association between the level of serum IL-6 and the number of sites with bleeding and PD ≥ 4 mm at the baseline and after periodontal therapy. The reduction in the number of sites with bleeding and PD ≥ 4 mm was lower in patients with higher serum IL-6 levels after periodontal therapy.	Subjects with a high IL-6 serum level after therapy presented poorer periodontal healing than those with a low level. IL-6 may have a modulatory effect on host immune response in T1DM patients.
Salvi et al., 2010 [73]	Prospective cohort study of experimental gingivitis (EG)	A total of 18 Caucasian subjects (9 patients with T1DM and 9 without diabetes) (age 25.6 ± 5.8 y.o.) were included. EG: Patients were instructed to refrain from all oralhygiene practices for 21 days, resuming oral hygiene practices following the 21-day exam, continuing for an additional 2 weeks until Day 35.	Periodontal exam and GCF was collected at baseline, Day 7, Day 14, Day 21 and Day 35. IL-1β, IL-8, MMP-8, and MMP-9 levels were determined by ELISA.	IL-1β levels in T1DM patients were higher compared to healthy individuals, and showed differences between groups at 7–21 days, while healthy patients showed IL-1b increases from baseline to 14–21 days (*p* < 0.05). MMP-9 levels between patients with and without T1DM at 7–14 days (*p* < 0.05).	GCF IL-1β and MMP-9 were most significantly elevated in T1DM subjects compared with healthy individuals during EG, not resulting from differences in the plaque index (PI) or microbial composition.
Aspriello et al., 2011 [74]	Cross-sectional study	Plasma C-reactive protein and GCF IL-1β, IL-6 and TNF-α were measured in periodontitis patients affected by T1DM (n = 24) and type 2 diabetes mellitus (T2DM) (n = 24). T1DM patients had a significantly lower age (43.5 ± 6.5 y.o) compared to T2DM ones (63.5 ± 15.5 y.o).	Plasma high-sensitive C-reactive protein (hs-CRP) concentrations were measured by a particle-enhanced immunoturbidimetric assay. GCF mediators were measured with ELISA.	No differences in any clinical periodontal parameters between T1DM and T2DM periodontitis patients. GCF IL-1β and TNF- α were higher in T1DM periodontitis patients compared to T2DM periodontitis ones. GCF IL-1β and TNF-α levels were negatively correlated with diabetes duration and are higher in cases of recent onset of diabetes mellitus.	GCF IL-1β and TNF-α levels were higher in T1DM periodontitis patients compared to T2DM periodontitis ones. GCF IL-1β and TNF-α levels higher in cases of recent onset of diabetes mellitus highlighting the need for periodontal therapy at the early stages of T1DM development.
Musial et al., 2021 [75]	Cross-sectional study	20 T1DM patients after simultaneous pancreas and kidney transplantation (SPK) and 16 after kidney transplantation (KTx), and 15 non-diabetic kidney recipients (control) were included. The minimal post-transplant follow-up period was 12 months.	GCF samples were collected and IL-1β, TNF-α, resistin, YKL-40 were measured with ELISA.	GCF concentration of all analyzed inflammation markers was lower in the SPK group than in the group of T1D patients after KTx (*p* < 0.001) Patients with T1DM after SPK showed lower GCF levels of all inflammatory markers and presented reduced intensity of periodontitis compared to kidney recipients treated with insulin but also to T1DM patients without any therapy.	Good metabolic control achieved by simultaneous pancreas and kidney transplantation (SPK) can decrease severity of periodontal inflammation in patients with end-stage renal disease caused by T1DM.
Lappin et al., 2009 [76]	Cross-sectional study	Plasma concentrations of receptor activator of nuclear factor-kB ligand (RANKL), osteoprotegerin (OPG), C-terminal telopeptide of type 1 collagen and osteocalcin were measured in T1DM patients (n = 63) and non-diabetics (n = 38). The age range of the subjects was 22–56 years.	Plasma levels of RANKL, OPG, C-terminal telopeptide of type 1 collagen and osteocalcin were measured with ELISA.	T1DM patients had significantly lower osteocalcin concentrations, lower RANKL to OPG ratios and higher OPG concentrations than non-diabetics. Osteocalcin had a negative correlation while OPG had a positive correlation with HbA1C.	T1DM patients with periodontitis showed lower serum osteocalcin and higher OPG levels than non-diabetic periodontitis patients, suggesting that T1DM patients have a decreased intrinsic ability to replace bone when that has been destroyed during ‘‘acute bursts’’ of periodontitis.
Chairatnathrongporn et al., 2022 [77]	Cross-sectional study	Twenty patients with T1DM and twenty age-matched non-T1DM patients participated (age: 18.35 ± 2.01 years). Periodontal exams and unstimulated whole saliva was collected.	RNA analysis of salivary samples for RANK, RANKL and OPG gene expression was conducted.	Patients with T1DM had more gingival inflammation and a higher percentage of full mouth gingival bleeding than the non-T1DM group, while plaque index (PI) was not significantly different. The relative mean mRNA levels of the RANK and RANKL genes, and the RANKL:OPG ratio demonstrated higher in the T1DM group than in the non-T1DM group.	More studies aiming to understand the role of bone metabolism by exploring bone markers are needed in patients with T1DM and periodontal diseases.

T1DM: type 1 diabetes mellitus; GCF: gingival crevicular fluid; HbA1C: hemoglobin A1C; BOP: bleeding on probing.

## Data Availability

All data generated or analyzed for this review are included in this published article. They are also available on request from the corresponding author (A.Z.).
